# Perfluorooctanoic Acid Exposure and Cancer Outcomes in a Contaminated Community: A Geographic Analysis

**DOI:** 10.1289/ehp.1205829

**Published:** 2013-01-08

**Authors:** Verónica M. Vieira, Kate Hoffman, Hyeong-Moo Shin, Janice M. Weinberg, Thomas F. Webster, Tony Fletcher

**Affiliations:** 1 Department of Environmental Health, Boston University School of Public Health, Boston, Massachusetts, USA; 2 Program in Public Health, Chao Family Comprehensive Cancer Center, University of California, Irvine, Irvine, California, USA; 3 Gillings School of Global Public Health, University of North Carolina at Chapel Hill, Chapel Hill, North Carolina, USA; 4 School of Social Ecology, University of California, Irvine, Irvine, California, USA; 5 Department of Public Health Sciences, University of California, Davis, Davis, California, USA; 6 Department of Biostatistics, Boston University School of Public Health, Boston, Massachusetts, USA; 7 Department of Social and Environmental Health Research, London School of Hygiene and Tropical Medicine, London, United Kingdom

**Keywords:** C8, GIS, kidney cancer, PFOA, testicular cancer

## Abstract

Background: Perfluorooctanoic acid (PFOA) has been linked to cancer in occupational mortality studies and animal toxicologic research.

Objective: We investigated the relationship between PFOA exposure and cancer among residents living near the DuPont Teflon-manufacturing plant in Parkersburg, West Virginia (WV).

Methods: Our analyses included incident cases of 18 cancers diagnosed from 1996 through 2005 in five Ohio (OH) counties and eight WV counties. For analyses of each cancer outcome, controls comprised all other cancers in the study data set except kidney, pancreatic, testicular, and liver cancers, which have been associated with PFOA in animal or human studies. We applied logistic regression models to individual-level data to calculate adjusted odds ratios (AORs) and confidence intervals (CIs). For the combined analysis of OH and WV data, the exposure of interest was resident water district. Within OH, geocoded addresses were integrated with a PFOA exposure model to examine the relationship between cancer odds and categories of estimated PFOA serum.

Results: Our final data set included 7,869 OH cases and 17,238 WV cases. There was a positive association between kidney cancer and the very high and high serum exposure categories [AOR = 2.0 (95% CI: 1.0, 3.9) *n* = 9 and 2.0 (95% CI: 1.3, 3.2) *n* = 22, respectively] and a null association with the other exposure categories compared with the unexposed. The largest AOR was for testicular cancer with the very high exposure category [2.8 (95% CI: 0.8, 9.2) *n* = 6], but there was an inverse association with the lower exposure groups, and all estimates were imprecise because of small case numbers.

Conclusions: Our results suggest that higher PFOA serum levels may be associated with testicular, kidney, prostate, and ovarian cancers and non-Hodgkin lymphoma. Strengths of this study include near-complete case ascertainment for state residents and well-characterized contrasts in predicted PFOA serum levels from six contaminated water supplies.

This study is one of a series of studies investigating health effects of perfluorooctanoic acid (PFOA, or C8) exposure among residents living near the Washington Works DuPont Teflon-manufacturing plant in Parkersburg, West Virginia, USA (Steenland et al. 2009). We used geographic methods to investigate the relationship between exposure to PFOA and patterns of cancer risk in the mid-Ohio River Valley using data from the Ohio (OH) and West Virginia (WV) cancer registries. PFOA was released into the environment via aerial emissions and discharged into the Ohio River beginning in the 1950s, resulting in the contamination of the local drinking water (Paustenbach et al. 2007; Shin et al. 2011a). In addition to hundreds of affected private drinking-water wells, six nearby public water districts in OH and WV were also contaminated (Figure 1), and monitoring data show that even after a drastic reduction in releases, PFOA contamination of drinking water persisted and continued to increase in some water districts near the plant (Shin et al. 2012).

**Figure 1:**
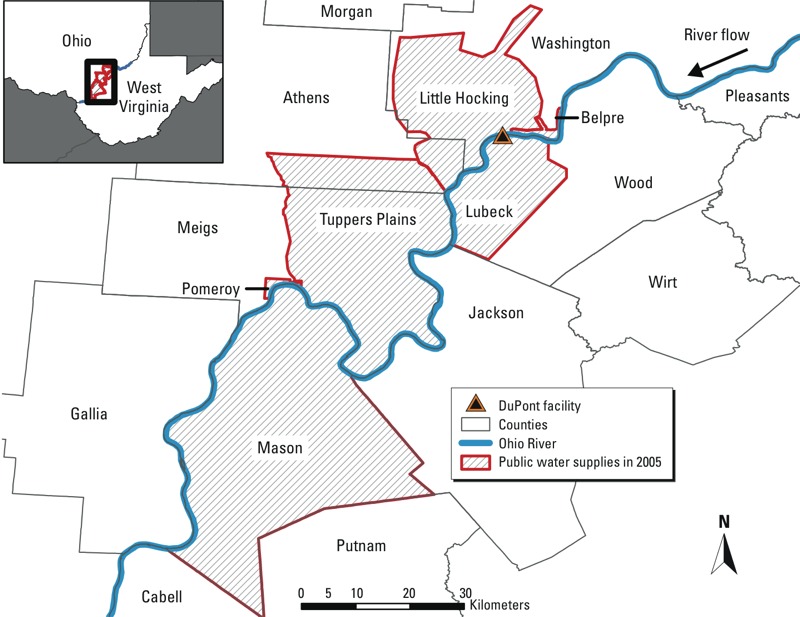
Study area of 13 counties encompassing six contaminated water districts.

As part of a settlement from a large class action lawsuit against DuPont, the C8 Science Panel (2012) was established to investigate potential health effects resulting from PFOA exposure, and a 1-year cross-sectional survey (2005–2006) known as the C8 Health Project was conducted among > 69,000 residents with ≥ 1-year residency in public water districts contaminated by PFOA (Frisbee et al. 2009). Measured mean PFOA levels in public drinking-water supplies at the time of the survey ranged from 0.03 µg/L in Mason, WV, to 3.49 µg/L in Little Hocking, OH, and in private drinking water, PFOA was measured at levels of ≤ 22.1 µg/L (Shin et al. 2011a). The median serum PFOA level in this cross-sectional study population was 28.2 µg/L, with a range of 0.2–22,412 µg/L (Steenland et al. 2009). PFOA has also been detected in the serum of the general U.S. population, albeit at a much lower median level of 3.9 µg/L (Calafat et al. 2007).

PFOA is widely used because of its stain-resistant and water-repellant properties, and given its persistence, it is ubiquitous in many indoor environments, including homes and workplaces (Fraser et al. 2012; Haug et al. 2011). Animal toxicologic data links PFOA to pancreatic cancer (acinar cells), testicular cancer (Leydig cells), and liver cancer (Lau et al. 2007). A recent review of the epidemiologic data concluded that more studies were needed to determine whether any potential health effects exist and, specifically, that the evidence for cancer is not conclusive (Steenland et al. 2010). Human data for cancer from two occupational cohorts are limited to mortality and are based on small numbers. One of the two cohorts showed an excess of kidney cancer (Leonard et al. 2008), and the other showed positive exposure–response trends for pancreatic and prostate cancer that were not statistically significant (Lundin et al. 2009). In a prospective Danish cohort study, plasma concentrations of background PFOA exposures were not associated with prostate, bladder, pancreatic, or liver cancer (Eriksen et al. 2009). A case–control study of Greenland Inuit women found a positive but not statistically significant association between PFOA exposure and breast cancer (Bonefeld-Jorgensen et al. 2011). The positive associations were generally not consistent among cancer sites between studies, and for the remaining cancer sites reported, no associations were observed.

The objective of this study was to investigate the association between PFOA exposure and the odds of cancer using data from the OH and WV cancer registries. The present study included residents of PFOA-exposed water districts and unexposed geographic areas outside of the C8 Health Project area, enabling a comparison between populations exposed to varying degrees and unexposed populations while controlling for individual-level risk factors. Results of this geographic study complement other studies being done within the C8 Health Project population by including a more complete ascertainment of cases, including those who died prior to the 2005–2006 survey. The weight of evidence from the combination of studies within this population was heavily considered in the determination by the C8 Science Panel that there was a probable link between PFOA exposure and testicular and kidney cancers.

## Methods

*Study population and data.* The study area encompasses six contaminated public water districts (WDs) and 13 counties in OH and WV that surround the Washington Works DuPont facility (Figure 1). Incident cancer cases diagnosed from 1996 through 2005 in the OH counties of Athens, Meigs, Gallia, Washington, and Morgan and the WV counties of Wood, Mason, Wirt, Putnam, Jackson, Pleasants, Ritchie, and Cabell were obtained from the OH Cancer Incidence Surveillance System (OCISS) and from the WV Cancer Registry (WVCR), respectively. The WVCR provided an incident cancer data set of all cancer types with a total of 19,716 individual cases. There were 10,044 (51%) male cases and 9,673 (49%) female cases. The OCISS provided us with registry data for 9,402 incident cases of all cancer types. For our analyses, we were able to geocode 8,650 (92%) of the OH addresses at diagnosis to the street level and the remaining 752 (8%) at the ZIP code level, with little variation in these proportions by cancer type. There were 4,396 (51%) male cases and 4,254 (49%) female cases. The median age for both data sets was 67 years. We excluded 745 OH cases and 2,411 WV cases of cancer types including oral cavity, pharynx, esophagus, larynx, stomach, and Hodgkin lymphoma with too few cases (< 100 OH cases, the smaller of the two analyses) for meaningful analysis, or that had not been previously investigated in relation to PFOA in animal toxicologic studies or occupational mortality studies (Lau et al. 2007; Leonard et al. 2008; Steenland and Woskie 2012). We also excluded 36 OH cases and 67 WV cases that were diagnosed at < 15 years of age. Our final data set included 7,869 geocoded OH cases and 17,238 WV cases of 18 cancer categories (i.e., bladder, brain, female breast, cervix, colon/rectum, kidney, leukemia, liver, lung, melanoma of the skin, multiple myeloma, non-Hodgkin lymphoma, ovary, pancreas, prostate, testis, thyroid, and uterus).

Based on 2010 U.S. census population estimates, the population of the study area is over 500,000, with one-third in OH and two-thirds in WV. Using a PFOA exposure model and data collected from the C8 Health Project, the corresponding 1995 median PFOA serum concentrations in the six public WDs were previously estimated as follows: Little Hocking (Washington and Athens Counties, OH), 125 µg/L; Lubeck (Wood County, WV), 65.8 µg/L; Tupper Plains (Athens and Meigs Counties, OH), 23.9 µg/L; Belpre (Washington County, OH), 18.7 µg/L; Pomeroy (Meigs County, OH), 10.7 µg/L; and Mason (Mason County, WV), 5.3 µg/L (Shin et al. 2011b). The institutional review boards at the Boston University Medical Campus, the London School of Hygiene and Tropical Medicine, the OH Department of Health, and the WV Bureau of Health Statistics approved the research. This study was granted a waiver of informed consent.

*Overview of analyses.* The final data set included information for study area residents diagnosed with 18 different categories of cancer. We applied logistic regression to individual-level data using registry-based cancer controls to calculate adjusted odds ratios (AORs) and confidence intervals (CIs) for each cancer category, with the other cancer categories excluding kidney, pancreatic, testicular, and liver cancers (which have been linked to PFOA exposure in animal and human studies previously) serving as controls. As a sensitivity analysis, we also performed analyses using a control group consisting of those persons with all other cancer diagnoses included in the data set, without exclusions. We adjusted for age, sex, diagnosis year, smoking status (current, past, unknown, with never smoker as the reference) and insurance provider (government-insured Medicaid, uninsured, unknown, with privately insured as the reference). We ran additional analyses stratified by sex for cancers with ≥ 100 cases of each sex; this included cancers of the bladder, colon/rectum, kidney, and lung, as well as melanoma of the skin and non-Hodgkin lymphoma. To test the sensitivity of results to missing smoking and health insurance data, we generated 10 data sets, with imputation of missing values using default predictive mean matching and logistic regression imputation via the “mice” library in R (van Buuren and Groothuis-Oudshoorn 2011). We obtained parameter estimates by averaging over all 10 data sets of parameter estimates, and variance estimates by combining the between- and within-imputation variances.

For exposure assessment purposes, the OCISS provided addresses at diagnosis that we geocoded, while the WVCR provided an identifier for each geographic unit, which allowed us to assign case addresses to contaminated water district areas or to the unexposed group. We conducted two different analyses to compare the robustness of our results across different exposure metrics. The first analysis used water district of residence as the exposure of interest and included both OH and WV data. The second analysis was restricted to OH and took advantage of the availability of geocoded OH addresses at time of diagnosis. We used an existing PFOA exposure model (Shin et al. 2011a, 2011b) to estimate serum levels at a finer geographic resolution for different latency assumptions. For OH-only analyses, we also adjusted for race, modeled as a binary variable for white or non-white, which was provided by the OCISS, but not the WVCR because of confidentiality concerns. The two analyses are described in detail below. All statistical analyses were conducted using R 2.10.1 (R Foundation, Vienna, Austria).

*Water district analysis for OH and WV data.* For the combined OH and WV data, we used residency within a contaminated water district area as our exposure of interest. For the OH data, we assigned cases to water districts using geocoding, the process by which measures of longitude and latitude are calculated for street addresses using reference street files. We first cleaned and standardized addresses using ZP4 address correction software with the LACS (Locatable Address Conversion System) database (version expiring 1 April 2011; Semaphore Corporation, Monterey, CA, USA) and then converted additional rural route boxes to street addresses using Enhanced 911 address conversion tables (Vieira et al. 2010). Geocoding was then performed using a geographic information system (GIS), ESRI ArcView version 9.3 (Redlands, CA, USA) with the ESRI StreetMap Premium North America NAVTEQ 2010 enhanced street data set as the reference address locator. Using geocoding, we were able to identify cases living within a contaminated water district area. Cases not in contaminated water districts were assigned to the unexposed group.

For WV cases, data release restrictions prohibited identifiable geographic location from being included with the cancer data. Instead, a variable was provided to indicate whether cases were located in Lubeck WD, Mason County WD, or unexposed areas. Only addresses in Wood County were geocoded to the water district distribution system at the WVCR to determine whether the case was living at a street address serviced by the Lubeck WD. Wood County cases not on the Lubeck WD distribution system were considered unexposed. All cases in Mason County were assigned exposure to the Mason County WD. Mason County addresses were not geocoded because the median PFOA serum levels were close to background.

We calculated AORs and CIs for each of the 18 cancer categories in association with one of the six contaminated water districts versus an unexposed water district. We also calculated the AORs for residence in any exposed water district relative to unexposed water districts.

*Estimated PFOA serum level analysis in OH.* To take advantage of the availability of geocoded street addresses in the OH data, we also used modeled serum PFOA concentration as an exposure metric. All OH addresses at time of diagnosis were geocoded to determine whether the case was serviced by one of the contaminated public water districts or a contaminated private well or was unexposed. This geocoding allowed us to be even more specific about exposure as cases living within a water district area, but not on a street serviced by a distribution pipe (or before the year of pipe installation), would likely be accessing drinking water from a private residential well. The methods for estimating individual serum PFOA levels from linked environmental, exposure, and pharmacokinetic models are described in detail elsewhere (Shin et al. 2011a, 2011b). Briefly, the environmental models integrate facility emissions data, fate and transport characteristics of PFOA, and hydrogeological properties of the study area to estimate PFOA air and water concentrations from 1951 through 2008. Using GIS, we were also able to determine what year the pipe that serviced each case was installed. For each case, annual PFOA serum levels were calculated from 1951 to date of diagnosis by linking historical air and groundwater concentrations to residential information at time of diagnosis and applying standard assumptions about water intake, body weights, and a PFOA half-life in the exposure and pharmacokinetic models (Shin et al. 2011b). Because only the residence at diagnosis was available, annual serum levels were estimated assuming cases lived at that address for 10 years. As a sensitivity analysis, we also estimated serum levels with and without a 10-year latency period prior to date of diagnosis, assuming a lifetime residency at that address. We then extracted two exposure metrics for each latency and residency assumption: *a*) the estimated annual serum level corresponding to the year of diagnosis or 10 years prior for latency analyses, and *b*) a cumulative measure summed over the corresponding years of exposure. The estimated annual serum level is equivalent to what would be measured in a serum sample taken during that year, whereas the cumulative measure is the area under the serum level profile curve.

We first categorized individual-level exposure as very high, high, medium, low, and unexposed using cutoffs based on the distribution of the annual PFOA serum concentrations among the exposed study population, assuming a 10-year residency. The distribution of estimated annual PFOA serum levels among the exposed study population ranged from 3.7 to 655 µg/L for 10-year residency, assuming 10-year latency [see Supplemental Material, Figure S1 (http://dx.doi.org/10.1289/ehp.1205829)]. The tertile breaks of the distribution defined the cutoffs for low, medium, and high. We used the tertile breaks of 12.9 and 30.8 to define high (30.8–109 µg/L), medium (12.9–30.7 µg/L), and low exposure categories (3.7–12.9), with unexposed serving as the reference category. There was a large break in the distribution at 110 µg/L, so a very high group was created based on this break value that included the upper 10% of our exposed population (see Supplemental Material, Figure S1). Cumulative exposure categories were based on the distribution among the exposed cases and were divided into the following groups: very high = 600–4,679 µg/L-years; high = 198–599 µg/L-years; medium = 89–197 µg/L-years; and low = 3.9–88 µg/L-years. We analyzed the individual-level OH data using logistic regression to calculate AORs and CIs for exposure categories, with unexposed serving as the referent. For comparison, separate analyses were conducted for the annual and cumulative exposure measures calculated for the different latency and residency assumptions.

## Results

*OH and WV water district analyses.* Table 1 shows the distribution of cases in the contaminated water district areas and the surrounding unexposed geographic area. The Little Hocking WD is the highest exposed district, followed by Lubeck, Tuppers Plains, Belpre, Pomeroy, and Mason County. The odds (AORs) of testicular cancer were increased in Little Hocking [5.1 (95% CI: 1.6, 15.6); *n* = 8], and the odds of kidney cancer was elevated in Little Hocking [1.7 (95% CI: 0.4, 3.3; *n* = 10)] and Tuppers Plains [2.0 (95% CI: 1.3, 3.1) *n* = 23]. Residents of Little Hocking also had increased odds of non-Hodgkin lymphoma [1.6 (95% CI: 0.9, 2.8) *n* = 14] and prostate cancer [1.4 (95% CI: 0.9, 2.30 *n* = 36].

**Table 1:** WV and OH water district results: *n* and AORs (95% CIs) for exposure to contaminated water districts.

Cancer outcome	Total	Total exposed		Little Hocking		Lubeck		Tuppers Plains		Belpre		Pomeroy		Mason	
	n	n	AOR (CI)	n	AOR (CI)	n	AOR (CI)	n	AOR (CI)	n	AOR (CI)	n	AOR (CI)	n	AOR (CI)
Bladder	1,350	137	0.8 (0.7, 1.0)	7	0.6 (0.3, 1.4)	24	1.0 (0.6, 1.5)	20	0.9 (0.6, 1.5)	24	1.1 (0.7, 1.6)	4	0.8 (0.3, 2.1)	58	0.7 (0.6, 1.0)
Brain	506	60	1.0 (0.8, 1.3)	1	0.2 (0.0, 1.5)	7	0.8 (0.4, 1.8)	9	1.1 (0.5, 2.1)	11	1.2 (0.6, 2.2)	3	1.7 (0.5, 5.4)	29	1.1 (0.7, 1.6)
Female breast	4,057	436	1.0 (0.9, 1.1)	33	1.2 (0.8, 2.0)	69	1.2 (0.9, 1.7)	50	0.7 (0.5, 1.1)	73	1.1 (0.8, 1.5)	18	0.8 (0.5, 1.5)	193	1.0 (0.8, 1.2)
Cervix	338	35	0.8 (0.6, 1.2)	4	0.9 (0.3, 2.9)	5	0.7 (0.3, 1.7)	8	1.8 (0.8, 3.8)	5	0.6 (0.2, 1.6)	2	0.9 (0.2, 4.1)	11	0.7 (0.4, 1.3)
Colon/rectum	3,543	383	0.9 (0.8, 1.0)	20	0.7 (0.5, 1.2)	44	0.7 (0.5, 1.0)	66	1.2 (0.9, 1.6)	55	0.9 (0.7, 1.2)	18	1.2 (0.7, 2.1)	180	0.9 (0.8, 1.1)
Kidney	751	94	1.1 (0.9, 1.4)	10	1.7 (0.9, 3.3)	9	0.7 (0.4, 1.3)	23	2.0 (1.3, 3.1)	17	1.4 (0.8, 2.3)	0	—	35	0.9 (0.6, 1.3)
Leukemia	674	72	0.9 (0.7, 1.1)	5	1.0 (0.4, 2.3)	11	0.9 (0.5, 1.6)	9	0.8 (0.4, 1.7)	12	1.0 (0.6, 1.9)	1	0.4 (0.1, 2.8)	34	0.9 (0.6, 1.3)
Liver	179	23	1.1 (0.7, 1.6)	1	0.8 (0.1, 5.6)	4	1.3 (0.5, 3.5)	3	1.0 (0.3, 3.3)	3	1.0 (0.3, 3.1)	1	1.4 (0.2, 10.5)	11	1.0 (0.5, 1.9)
Lung	4,926	632	1.2 (1.1, 1.3)	37	1.0 (0.7, 1.5)	85	1.1 (0.8, 1.4)	84	1.3 (1.0, 1.7)	90	1.1 (0.9, 1.4)	23	1.1 (0.7, 1.8)	313	1.3 (1.1, 1.5)
Melanoma of the skin	1,428	168	0.9 (0.8, 1.1)	12	1.0 (0.6, 1.9)	32	1.2 (0.8, 1.7)	21	0.9 (0.6, 1.4)	38	1.4 (1.0, 2.0)	4	0.9 (0.3, 2.5)	61	0.7 (0.5, 0.9)
Multiple myeloma	285	36	1.1 (0.8, 1.6)	1	0.5 (0.1, 3.6)	4	0.9 (0.3, 2.3)	3	0.7 (0.2, 2.2)	7	1.5 (0.7, 3.2)	1	0.9 (0.1, 6.6)	20	1.4 (0.9, 2.2)
Non-Hodgkin lymphoma	1,124	152	1.2 (1.0, 1.5)	14	1.6 (0.9, 2.8)	20	1.1 (0.7, 1.7)	21	1.2 (0.8, 1.9)	24	1.3 (0.9, 2.0)	5	1.1 (0.4, 2.7)	68	1.2 (0.9, 1.5)
Ovary	417	48	1.0 (0.8, 1.4)	5	1.8 (0.7, 4.4)	5	0.7 (0.3, 1.7)	6	1.1 (0.5, 2.4)	11	1.6 (0.9, 3.0)	2	1.1 (0.3, 4.4)	19	0.9 (0.5, 1.4)
Pancreas	495	58	1.0 (0.8, 1.3)	4	1.1 (0.4, 3.0)	9	1.1 (0.6, 2.1)	10	1.3 (0.7, 2.5)	8	0.9 (0.4, 1.8)	2	1.0 (0.2, 4.1)	25	0.9 (0.6, 1.4)
Prostate	3,678	434	0.9 (0.8, 1.1)	36	1.4 (0.9, 2.3)	78	1.2 (0.9, 1.6)	56	0.8 (0.6, 1.1)	56	0.8 (0.6, 1.1)	12	1.3 (0.6, 2.6)	196	0.9 (0.7, 1.0)
Testis	134	18	1.0 (0.6, 1.8)	8	5.1 (1.6, 15.6)	2	0.9 (0.2, 4.5)	2	0.4 (0.1, 2.0)	1	0.6 (0.1, 5.0)	0	—	5	0.5 (0.2, 1.5)
Thyroid	343	40	1.1 (0.7, 1.5)	3	0.8 (0.3, 2.7)	7	1.2 (0.6, 2.6)	2	0.3 (0.1, 1.4)	5	0.9 (0.4, 2.2)	0	—	23	1.4 (0.9, 2.2)
Uterus	879	97	1.0 (0.8, 1.3)	7	1.1 (0.5, 2.4)	15	1.1 (0.6, 1.9)	12	0.9 (0.5, 1.6)	14	0.9 (0.5, 1.6)	4	0.9 (0.3, 2.4)	45	1.1 (0.8, 1.5)
Adjusted for age, sex, diagnosis year, insurance provider, and smoking status; controls were other listed cancers excluding kidney, liver, pancreas, and testis cancers. Estimated 1995 median PFOA serum concentrations in the WDs: Little Hocking = 125 µg/L; Lubeck = 65.8 µg/L; Tupper Plains = 23.9 µg/L; Belpre = 18.7 µg/L; Pomeroy = 10.7 µg/L; and Mason = 5.3 µg/L; reference = unexposed.															

*OH serum level analyses.* AORs suggested associations between the very high PFOA exposure category and several cancers, but AORs for lower exposure categories generally did not support a positive dose–response relation (Table 2). Kidney cancer was positively associated with very high and high exposure categories [2.0, (95% CI: 1.0, 3.9) *n* = 9 and 2.0 (95% CI: 1.3, 3.2) *n* = 22, respectively], whereas AORs for medium and low exposure categories were close to the null compared with the unexposed. The largest AOR was for testicular cancer with the very high exposure category [2.8 (95% CI: 0.8, 9.2) *n* = 6] but the estimate was imprecise due to small numbers, and AORs for high, medium, or low exposure categories, which were based on only 1, 3, and 1 cases, respectively, were all < 1.0. Ovarian cancer was also positively associated with the very high exposure category [2.1 (95% CI: 0.8, 5.5) *n* = 5] but again imprecise because of small numbers, and weaker associations for the high and medium exposure categories with a negative association in the lowest exposure category. AORs for the association between the very high and medium exposure categories and non-Hodgkin lymphoma were moderate [1.8 (95% CI: 1.0, 3.4) *n* = 11 and 1.5 (95% CI: 1.0, 2.2) *n* = 28, respectively], while AORs for high and low exposure categories were close to the null compared with the unexposed. Prostate cancer showed a weak but relatively precise positive association with very high exposure [1.5 (95% CI: 0.9, 2.5) *n* = 31] and no association with lower levels of exposure. Results were very similar for associations with the cumulative exposure measure [see Supplemental Material, Table S1 (http://dx.doi.org/10.1289/ehp.1205829)], and for exposure estimates that did not account for latency (see Supplemental Material, Table S2), which were highly correlated with estimated exposures that assumed a 10-year latency (Spearman’s rank correlation *r*_S_ = 0.997, *p* < 0.001). In addition, associations were similar when the alternative control group (which included kidney, liver, pancreas, and testis cancer cases) was used (see Supplemental Material, Table S3).

**Table 2:** OH serum-level results: *n* and AORs (95% CIs) for individual-level annual PFOA serum exposure categories assuming 10-year residency and latency.

Cancer outcome	Total	Total exposed	Very high		High		Medium		Low	
	n	n	n	AOR (CI)	n	AOR (CI)	n	AOR (CI)	n	AOR (CI)
Bladder	395	69	4	0.6 (0.2, 1.5)	21	1.2 (0.8, 2.0)	21	0.9 (0.6, 1.4)	23	0.9 (0.6, 1.4)
Brain	150	32	0	—	4	0.6 (0.2, 1.6)	16	1.8 (1.1, 3.2)	12	1.5 (0.8, 2.7)
Female breast	1,260	223	29	1.4 (0.9, 2.3)	45	0.7 (0.5, 1.0)	77	1.1 (0.8, 1.5)	72	0.9 (0.7, 1.2)
Cervix	144	25	2	0.6 (0.1, 2.6)	8	1.7 (0.8, 3.8)	4	0.5 (0.2, 1.5)	11	1.1 (0.6, 2.2)
Colon/rectum	1,149	212	13	0.6 (0.3, 1.0)	63	1.3 (1.0, 1.7)	64	0.9 (0.7, 1.2)	72	1.0 (0.8, 1.3)
Kidney	246	59	9	2.0 (1.0, 3.9)	22	2.0 (1.3, 3.2)	17	1.2 (0.7, 2.0)	11	0.8 (0.4, 1.5)
Leukemia	191	36	2	0.6 (0.1, 2.3)	8	0.9 (0.4, 1.8)	12	1.0 (0.6, 1.9)	14	1.2 (0.7, 2.1)
Liver	61	11	0	—	3	1.0 (0.3, 3.1)	4	0.9 (0.3, 2.5)	4	1.1 (0.4, 3.1)
Lung	1,526	293	29	1.0 (0.7, 1.6)	78	1.2 (0.9, 1.6)	95	1.0 (0.8, 1.3)	91	1.0 (0.7, 1.2)
Melanoma of the skin	429	95	9	0.9 (0.5, 1.9)	21	1.0 (0.6, 1.5)	38	1.3 (0.9, 1.8)	27	1.2 (0.8, 1.8)
Multiple myeloma	83	18	1	0.6 (0.1, 4.7)	4	1.0 (0.3, 2.7)	6	1.1 (0.5, 2.6)	7	1.4 (0.7, 3.2)
Non-Hodgkin lymphoma	347	76	11	1.8 (1.0, 3.4)	17	1.1 (0.7, 1.9)	28	1.5 (1.0, 2.2)	20	1.0 (0.6, 1.6)
Ovary	128	27	5	2.1 (0.8, 5.5)	8	1.4 (0.7, 2.9)	10	1.4 (0.7, 2.7)	4	0.5 (0.2, 1.4)
Pancreas	162	33	2	0.6 (0.1, 2.5)	9	1.1 (0.6, 2.3)	10	0.9 (0.5, 1.7)	12	1.3 (0.7, 2.3)
Prostate	1,155	214	31	1.5 (0.9, 2.5)	47	0.8 (0.5, 1.1)	65	0.8 (0.6, 1.0)	71	1.1 (0.8, 1.5)
Testis	61	11	6	2.8 (0.8, 9.2)	1	0.3 (0.0, 2.7)	3	0.6 (0.2, 2.2)	1	0.2 (0.0, 1.6)
Thyroid	94	15	2	0.8 (0.2, 3.5)	3	0.7 (0.2, 2.1)	5	0.9 (0.4, 2.3)	5	0.9 (0.4, 2.3)
Uterus	288	47	4	0.7 (0.3, 1.5)	12	1.7 (1.2, 2.5)	14	0.9 (0.6, 1.3)	17	1.2 (0.8, 1.7)
Adjusted for age, race, sex, diagnosis year, insurance provider, and smoking status; controls were other listed cancers excluding kidney, liver, pancreas, and testis cancers. Categories of modeled PFOA serum concentrations: very high = 110–655 µg/L; high = 30.8–109 µg/L; medium = 12.9–30.7 µg/L; low = 3.7–12.8 µg/L; reference = unexposed.										

To test the sensitivity of our analyses to missing smoking (*n* = 2,452) and health insurance data (*n* = 1,824), we ran multiple imputations for cancers of the bladder, colon/rectum, female breast, kidney, lung, prostate and uterus and melanoma of the skin and non-Hodgkin lymphoma with sufficient numbers (≥ 100 cases with complete covariate data) and we observed similar results [see Supplemental Material, Table S4 (http://dx.doi.org/10.1289/ehp.1205829)].

For cancers of the bladder, colon/rectum, kidney, and lung and melanoma of the skin and non-Hodgkin lymphoma with sufficient numbers to stratify by sex (≥ 100 cases in men and women, respectively), we observed generally similar results with regard to PFOA exposure categories (data not shown). An exception was kidney cancer, which was positively associated with very high exposure in women (*n* = 108) [AOR = 3.5 (95% CI: 1.4, 8.3) *n* = 6] but not men (*n* = 138) [AOR = 1.0 (95% CI: 0.3, 3.4) *n* = 3] compared with the unexposed.

## Discussion

Testicular cancer was positively associated with the highest PFOA water district [AOR = 5.1 (95% CI: 1.6, 15.6) *n* = 8] and the highest serum exposure category [AOR = 2.8 (95% CI: 0.8, 9.2) *n* = 6] compared with cases living in unexposed areas. However, we also observed an inverse association between testicular cancer and the lower exposure groups, and all of the estimates were imprecise because of small numbers of cases. Evidence of effects of PFOA on testicular Leydig cell tumors in animal models has been reported (Lau et al. 2007). Kidney cancer was increased in association with both high and very high PFOA exposure, based on larger numbers of cases. We also observed elevated AORs for very high PFOA exposure and ovarian [2.0 (95% CI: 0.8, 5.1) *n* = 5] and prostate [1.5 (95% CI: 1.0, 2.5) *n* = 31] cancers and non-Hodgkin lymphoma [1.8 (95% CI: 0.9, 3.3) *n* = 11].

A limitation of our study is that we used other types of cancer as our controls (referents). In our analysis we assumed that referent cancers were not associated with exposure to PFOA. For our main analyses we excluded kidney, pancreatic, testicular, and liver cancers from controls because these cancers have been linked to PFOA exposure in animal or human studies previously; however, analyses using all other cancers as referents were comparable. We further assumed that different types of cancer were ascertained by the registry in the same way, and that they were sampled from the same source population.

Despite the large overall sample size of our study, the water district analyses and the analyses of the very high exposure group were limited by small numbers of many individual cancer cases. There was also little consistency in the results across exposure categories, with no evidence of a positive dose response. We were further limited by the covariates we could adjust for, which included only age, sex, race (white or non-white, OH only), smoking status, and health insurance provider. We were therefore unable to adjust for other risk factors of potential interest (e.g., prenatal exposure to xenoestrogens in relation to testicular cancer) although such factors would also have to have been associated with exposure to cause confounding. Chance is also a concern because we are investigating multiple cancer sites.

As expected under the assumption that a positive association is truly present, we observed similar but weaker associations for most outcomes when no latency was assumed. Because exposure in our study was dependent on location and the ranking of exposure between districts generally remained stable over time, there was very little movement of cases across exposure categories with respect to latency assumptions. As a sensitivity analysis, we also modeled exposures assuming the cases lived at their residences their entire lifetimes and observed similar results [see Supplemental Materials, Table S4 (http://dx.doi.org/10.1289/ehp.1205829)]. However, both the latency and residential history measures were highly correlated (Spearman’s rank correlations: *r*_S_ > 0.99, *p* < 0.001). Moving within the same public water district would also have little or no impact on the estimated serum values, but moving across water districts, or especially from more distant locations, could lead to exposure misclassification. Based on data from the C8 Health Project for residents > 50 years of age, the median residency duration for their current residence in 2005–2006 was 17 years. Therefore, we felt the 10-year residency duration with a 10-year latency was a reasonable assumption. Any resulting exposure misclassification is likely nondifferential, so the bias in the highest exposure category should, on average, be toward the null.

Strengths of our study include a relatively large overall sample size, ascertainment of cases from cancer registries using controls from the same population as the cases, good success in geocoding of OH residences, and use of a validated exposure model for predicting serum levels. Previous work has shown that the correlation between measured and predicted serum in 2005–2006 using this exposure model was 0.82 (Shin et al. 2011b). Water and serum concentrations in the more exposed areas were well above background, providing a larger exposure contrast compared with other cancer studies of PFOA in general populations. We found qualitatively similar results for testicular, kidney, ovarian and prostate cancers and non-Hodgkin lymphoma using the two different analyses; the robustness of these results is another strength.

Both analyses used individual-level outcome and risk factor information, but the first analysis used a group-level water district exposure measure so that both OH and WV data could be analyzed together. The second analysis used estimates of serum PFOA as the exposure of interest, allowing us to use geocoded residences to estimate exposure metrics based on points in time or cumulative measures, but only for OH cases. The second analysis had the advantages of being a fully individual-level design [eliminating the possible semi-ecologic bias of the other analysis (Webster 2007)] and using an exposure model previously validated as a predictor of serum levels in this context. However, there was still a potential for exposure misclassification in using residence at diagnosis. A disadvantage is that we were able to analyze only OH data because geocoded residences were not available for WV.

Associations between PFOA exposures and the same cancers have been reported in other unpublished C8 Science Panel (2012) studies of the same community. Interviews of 32,254 adult community residents and DuPont workers were conducted in 2008–20011, and medical records were sought. Cox regression of hazard ratios of medically validated cancers in relation to modeled cumulative PFOA serum levels at dates of diagnoses indicated increasing kidney cancer risk with increasing exposure when latency was not considered. When 10-year latency was included in the exposure metric, the association was less evident. The relative risks (RRs) for testicular cancer in relation to increasing exposure quartiles with 10-year latency were 1.0, 1.2, 1.7, and 3.0. Cross-sectional analysis of prevalent cancers among 49,082 adult community members interviewed in 2005–2006 in relation to measured PFOA indicated increased RRs with increased serum PFOA quartiles compared with the lowest quartile (RRs = 1.0, 1.5, 1.7, and 1.7, respectively).

## Conclusions

The geographic analyses of cancer registry data provide some evidence that higher PFOA serum levels may be associated with certain cancers. The association in the highest PFOA exposure group was largest but very imprecise for testicular cancer, and smaller but more precise for kidney cancer. Non-Hodgkin lymphoma and ovarian and prostate cancers were associated with very high exposure based on some models, but there was little or no evidence of associations with other cancers. Analyses were limited by a case-only design with minimal control of confounders and by small case numbers despite having 10 years of data. In addition, residential history information was not available to account for latency, migration, and other issues regarding timing of exposure relative to cancer. However, the registries cover all residents in the study area, which comprises water districts with large, known contrasts in contamination. Thus, our geographic analyses using cancer registry data contributes to the evidence for the conclusion of the C8 Science Panel (2012) of a probable link between PFOA exposure and testicular and kidney cancers.

## Supplemental Material

(541 KB) PDF
