# Supplementary material for: Perfluorooctanoic Acid Exposure and Cancer Outcomes in a Contaminated Community: A Geographic Analysis
Source: Environ Health Perspect. 2013 Jan 8;121(3):318–23. doi: 10.1289/ehp.1205829 (PMC3621179; doi:10.1289/ehp.1205829)
Supplement: (541 KB) PDF [file ehp.1205829.s001.pdf]

Supplemental Material

Perfluorooctanoic Acid Exposure and Cancer Outcomes in a Contaminated Community: A Geographic Analysis

Verónica M. Vieira, Kate Hoffman, Hyeong-Moo Shin, Janice M. Weinberg, Thomas F. Webster, Tony Fletcher

Table of Contents:

Supplemental Material, Table S1, page 2. OH serum-level results: adjusted odds ratios and 95% confidence intervals for cumulative PFOA serum exposure assuming 10-yr residency and latency

Supplemental Material, Table S2, page 3. OH serum-level results: adjusted odds ratios and 95% confidence intervals for annual PFOA serum exposure assuming 10-yr residency and no latency

Supplemental Material, Table S3, page 4. OH serum-level results: adjusted odds ratios and 95% confidence intervals for annual PFOA serum exposure assuming 10-yr residency and latency with alternative control group

Supplemental Material, Table S4, page 5. OH serum-level results: adjusted odds ratios and 95% confidence intervals for annual PFOA serum exposure assuming 10-yr residency and latency with multiple imputations for missing data

Supplemental Material, Figure S1, page 6. Histogram of serum estimates assuming a 10-yr residency and latency among exposed OH study population

Supplemental Material, Table S1. OH serum-level results: adjusted odds ratios[a] and 95% confidence intervals for cumulative PFOA serum exposure[b] assuming 10-yr residency and latency

Category	Very High	High	Medium	Low
Bladder	0.6 (0.2, 1.7)	1.0 (0.6, 1.7)	1.1 (0.7, 1.6)	0.9 (0.6, 1.4)
Brain	---[c]	0.7 (0.3, 1.8)	1.7 (1.0, 2.9)	1.5 (0.8, 2.7)
Female Breast	1.4 (0.8, 2.3)	0.7 (0.5, 0.9)	1.1 (0.8, 1.5)	0.9 (0.7, 1.2)
Cervix	0.6 (0.1, 2.7)	1.7 (0.7, 3.9)	0.6 (0.2, 1.6)	1.1 (0.6, 2.2)
Colon/Rectum	0.6 (0.3, 1.1)	1.2 (0.9, 1.6)	1.0 (0.7, 1.3)	1.0 (0.8, 1.3)
Kidney	2.1 (1.1, 4.2)	2.0 (1.3, 3.2)	1.2 (0.7, 2.0)	0.8 (0.4, 1.5)
Leukemia	0.6 (0.1, 2.4)	1.4 (0.8, 2.6)	0.7 (0.4, 1.5)	1.1 (0.6, 2.0)
Liver	---[c]	1.0 (0.3, 3.2)	1.2 (0.5, 3.0)	0.9 (0.3, 2.8)
Lung	0.9 (0.6, 1.5)	1.2 (0.9, 1.6)	1.0 (0.8, 1.3)	1.0 (0.7, 1.2)
Melanoma of the skin	0.9 (0.4, 1.8)	1.2 (0.8, 1.9)	1.2 (0.8, 1.7)	1.1 (0.8, 1.7)
Multiple Myeloma	0.7 (0.1, 5.0)	0.7 (0.2, 2.3)	1.1 (0.5, 2.6)	1.7 (0.8, 3.6)
Non-Hodgkin's Lymphoma	2.0 (1.0, 3.7)	1.0 (0.6, 1.7)	1.5 (1.0, 2.2)	1.0 (0.6, 1.6)
Ovary	2.2 (0.9, 5.7)	1.7 (0.9, 3.4)	0.9 (0.4, 2.1)	0.7 (0.3, 1.6)
Pancreas	0.6 (0.2, 2.7)	1.3 (0.7, 2.5)	0.7 (0.3, 1.4)	1.4 (0.8, 2.4)
Prostate	1.5 (0.9, 2.5)	0.8 (0.6, 1.1)	0.8 (0.6, 1.0)	1.1 (0.8, 1.5)
Testis	2.8 (0.8, 9.6)	0.4 (0.0, 2.9)	0.4 (0.1, 1.8)	0.4 (0.1, 1.9)
Thyroid	0.9 (0.2, 3.7)	0.9 (0.3, 2.5)	0.7 (0.3, 2.0)	0.9 (0.4, 2.3)
Uterus	0.7 (0.3, 1.7)	1.6 (1.1, 2.3)	0.9 (0.6, 1.3)	1.2 (0.8, 1.7)

a. Adjusted for age, race, gender, insurance provider, and smoking status. Controls were other listed cancers excluding kidney, liver, pancreas and testis cancers.
a. Categories of modeled PFOA serum concentrations (µg/L-year): Very High=600-4679 µg/L-year; High: 198-599 µg/L-year; Medium=89-197 µg/L-year; Low=3.8-88 µg/L-year; reference: unexposed
b. No cases in exposure category

Supplemental Material, Table S2. OH serum-level results: adjusted odds ratios[a] and 95% confidence intervals for annual PFOA serum exposure[b] assuming 10 year residency and no latency

Category	Very High	High	Medium	Low
Bladder	0.7 (0.3, 1.6)	1.1 (0.7, 1.7)	1.0 (0.6, 1.6)	0.8 (0.5, 1.4)
Brain	---[c]	1.1 (0.6, 2.1)	1.7 (0.9, 3.1)	1.3 (0.6, 2.7)
Female Breast	1.2 (0.7, 1.9)	0.7 (0.5, 0.9)	1.1 (0.9, 1.5)	1.0 (0.7, 1.4)
Cervix	1.0 (0.3, 2.8)	1.8 (0.9, 3.5)	0.5 (0.2, 1.3)	0.9 (0.4, 2.2)
Colon/Rectum	0.7 (0.4, 1.1)	1.2 (0.9, 1.5)	1.0 (0.7, 1.3)	1.0 (0.7, 1.3)
Kidney	1.7 (0.9, 3.3)	1.8 (1.2, 2.8)	1.1 (0.6, 1.9)	0.9 (0.5, 1.8)
Leukemia	1.1 (0.4, 2.7)	0.9 (0.5, 1.7)	0.9 (0.5, 1.7)	1.2 (0.6, 2.4)
Liver	---[c]	0.9 (0.3, 2.6)	0.8 (0.2, 2.5)	1.3 (0.4, 4.2)
Lung	1.0 (0.7, 1.4)	1.2 (0.9, 1.5)	1.1 (0.8, 1.4)	0.9 (0.6, 1.2)
Melanoma of the skin	1.0 (0.5, 1.9)	1.0 (0.6, 1.4)	1.3 (0.9, 1.9)	1.2 (0.8, 2.0)
Multiple Myeloma	0.5 (0.1, 3.7)	0.9 (0.4, 2.3)	1.1 (0.5, 2.6)	1.9 (0.8, 4.3)
Non-Hodgkin's Lymphoma	1.8 (1.0, 3.1)	1.3 (0.9, 2.0)	1.3 (0.8, 2.0)	0.9 (0.5, 1.6)
Ovary	1.7 (0.7, 4.2)	1.7 (0.9, 3.1)	0.9 (0.4, 2.1)	0.6 (0.2, 1.8)
Pancreas	0.9 (0.3, 2.6)	1.1 (0.6, 2.1)	0.9 (0.4, 1.7)	1.2 (0.6, 2.5)
Prostate	1.3 (0.8, 2.0)	0.8 (0.6, 1.1)	0.7 (0.5, 0.9)	1.3 (0.9, 1.8)
Testis	2.2 (0.7, 6.6)	0.6 (0.2, 2.0)	0.3 (0.0, 2.9)	---[c]
Thyroid	1.0 (0.3, 3.4)	0.5 (0.2, 1.6)	0.8 (0.3, 2.3)	1.2 (0.5, 3.0)
Uterus	0.7 (0.3, 1.5)	1.6 (1.1, 2.3)	0.9 (0.6, 1.4)	1.1 (0.7, 1.7)

b. Adjusted for age, race, gender, diagnosis year, insurance provider, and smoking status. Controls were other listed cancers excluding kidney, liver, pancreas and testis cancers.
a. Categories of modeled PFOA serum concentrations (µg/L): Very High=110-655 µg/L; High=30.8-109 µg/L; Medium=12.9-30.7 µg/L; Low=3.7-12.8 µg/L; reference = unexposed
b. No cases in exposure category

Supplemental Material, Table S3. OH serum-level results: adjusted odds ratios[a] and 95% confidence intervals for annual PFOA serum exposure[b] assuming 10-yr residency and latency with alternative control group

Category	Very High	High	Medium	Low
Bladder	0.6 (0.2, 1.5)	1.2 (0.8, 1.9)	0.9 (0.6, 1.4)	0.9 (0.6, 1.4)
Brain	---[c]	0.6 (0.2, 1.6)	1.8 (1.1, 3.2)	1.5 (0.8, 2.7)
Female Breast	1.3 (0.8, 2.1)	0.7 (0.5, 0.9)	1.1 (0.8, 1.5)	0.9 (0.7, 1.2)
Cervix	0.6 (0.1, 2.6)	1.7 (0.8, 3.7)	0.5 (0.2, 1.5)	1.1 (0.6, 2.2)
Colon/Rectum	0.6 (0.3, 1.0)	1.2 (0.9, 1.6)	0.9 (0.7, 1.2)	1.0 (0.8, 1.3)
Kidney	1.9 (1.0, 3.9)	2.0 (1.3, 3.2)	1.2 (0.7, 2.0)	0.8 (0.4, 1.5)
Leukemia	0.5 (0.1, 2.2)	0.9 (0.4, 1.8)	1.0 (0.6, 1.9)	1.2 (0.7, 2.1)
Liver	---[c]	0.9 (0.3, 3.1)	0.9 (0.3, 2.6)	1.2 (0.4, 3.3)
Lung	1.0 (0.6, 1.5)	1.2 (0.9, 1.5)	1.0 (0.8, 1.2)	1.0 (0.8, 1.3)
Melanoma of the skin	0.8 (0.4, 1.6)	0.9 (0.6, 1.5)	1.3 (0.9, 1.8)	1.2 (0.8, 1.8)
Multiple Myeloma	0.6 (0.1, 4.6)	0.9 (0.3, 2.6)	1.1 (0.5, 2.6)	1.5 (0.7, 3.2)
Non-Hodgkin's Lymphoma	1.8 (0.9, 3.3)	1.1 (0.7, 1.8)	1.5 (1.0, 2.2)	1.0 (0.6, 1.6)
Ovary	2.0 (0.8, 5.1)	1.3 (0.6, 2.8)	1.4 (0.7, 2.7)	0.5 (0.2, 1.4)
Pancreas	0.6 (0.1, 2.3)	1.1 (0.6, 2.2)	0.9 (0.5, 1.7)	1.3 (0.7, 2.3)
Prostate	1.5 (1.0, 2.5)	0.7 (0.5, 1.0)	0.8 (0.6, 1.0)	1.1 (0.8, 1.5)
Testis	2.4 (0.7, 7.5)	0.3 (0.0, 2.9)	0.6 (0.2, 2.3)	0.2 (0.0, 1.5)
Thyroid	0.8 (0.2, 3.3)	0.7 (0.2, 2.1)	0.9 (0.4, 2.3)	0.9 (0.4, 2.3)
Uterus	0.6 (0.3, 1.5)	1.6 (1.1, 2.4)	0.9 (0.6, 1.3)	1.2 (0.8, 1.7)

a. Adjusted for age, gender, race, diagnosis year, insurance provider, and smoking status. Cases of the other 17 cancer types were used as controls.
b. Categories of modeled PFOA serum concentrations (µg/L): Very High=110-655 µg/L; High=30.8-109 µg/L; Medium=12.9-30.7 µg/L; Low=3.7-12.8 µg/L; reference = unexposed
c. No cases in exposure category

Supplemental Material, Table S4. OH serum-level results: adjusted odds ratios[a] and 95% confidence intervals for annual PFOA serum exposure[b] assuming 10-yr residency and latency with multiple imputations[c] for missing data

Category	Very High	High	Medium	Low
Bladder	0.4 (0.1, 1.7)	1.3 (0.7, 2.4)	0.8 (0.4, 1.5)	0.9 (0.5, 1.5)
Female Breast	1.2 (0.6, 2.3)	0.9 (0.5, 1.4)	0.9 (0.6, 1.4)	0.9 (0.6, 1.3)
Colon/Rectum	0.7 (0.3, 1.3)	1.1 (0.8, 1.7)	0.6 (0.4, 1.0)	1.1 (0.8, 1.5)
Kidney	2.0 (0.3, 3.3)	2.3 (1.3, 4.1)	0.9 (0.4, 2.0)	0.6 (0.3, 1.4)
Lung	1.2 (0.6, 2.2)	1.6 (1.1, 2.4)	1.3 (0.9, 1.9)	0.9 (0.7, 1.3)
Melanoma of the Skin	0.9 (0.6, 1.3)	1.0 (0.8, 1.3)	1.5 (1.3, 1.8)	1.1 (0.9, 1.4)
Non-Hodgkin's Lymphoma	2.1 (1.0, 4.5)	0.8 (0.4, 1.8)	1.6 (0.9, 2.8)	1.1 (0.7, 1.9)
Prostate	1.9 (1.0, 3.6)	0.6 (0.4, 1.1)	1.0 (0.7, 1.7)	1.3 (0.9, 1.9)
Uterus	0.9 (0.3, 3.0)	1.0 (0.5, 2.1)	0.6 (0.2, 1.6)	0.9 (0.5, 1.7)

c. Adjusted for age, race, gender, diagnosis year, insurance provider, and smoking status. Controls were other listed cancers excluding kidney, liver, pancreas and testis cancers.
d. Categories of modeled PFOA serum concentrations (µg/L): Very High=110-655 µg/L; High=30.8-109 µg/L; Medium=12.9-30.7 µg/L; Low=3.7-12.8 µg/L; reference = unexposed
e. Multiple imputations were only run for cancers with sufficient number of cases (≥ 100 cases with complete covariate information)

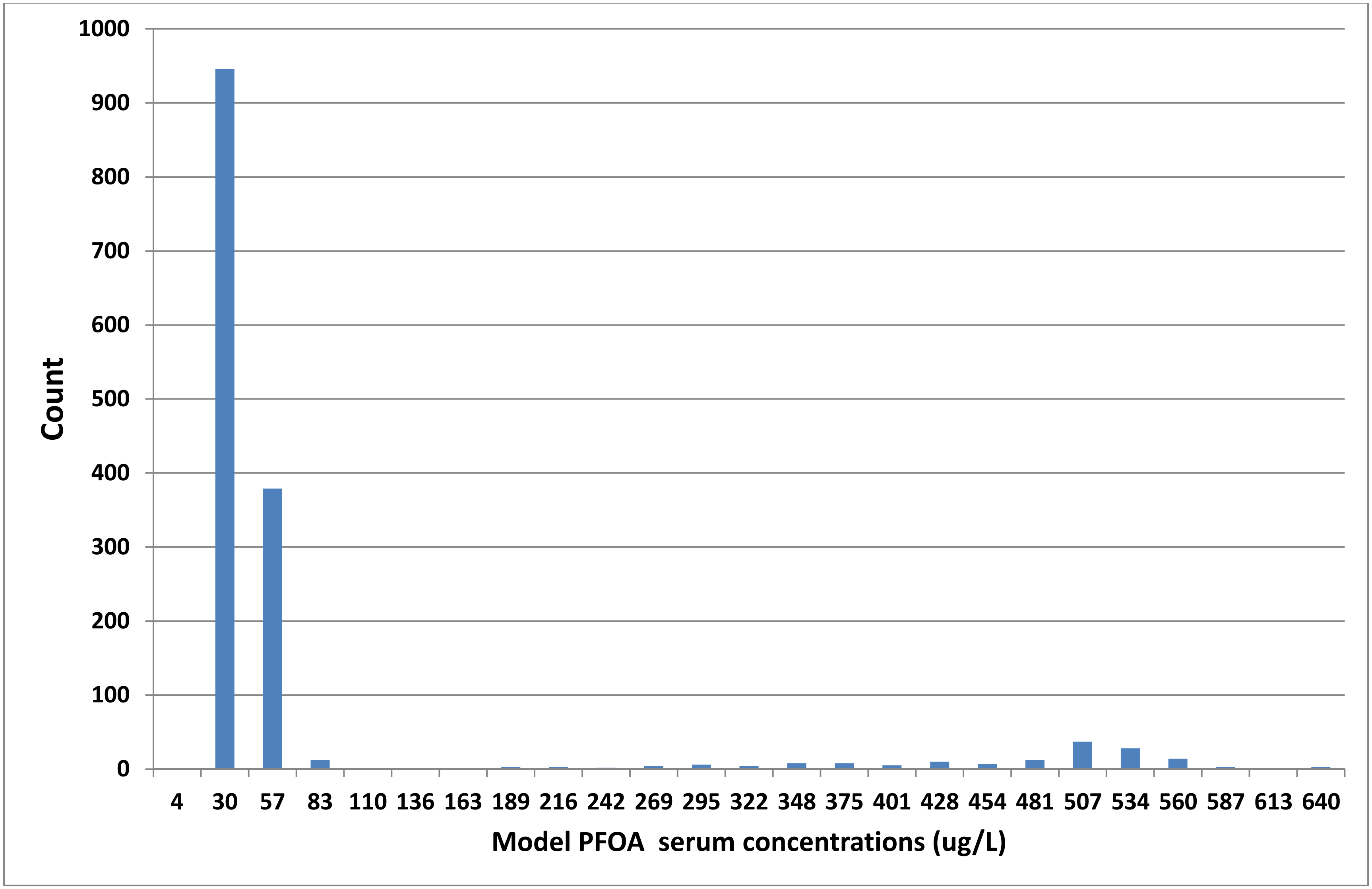

Supplemental Material, Figure S1. Histogram of serum estimates assuming a 10-yr residency and latency among the exposed OH study population
